# Gene-by-environment interactions that disrupt mitochondrial homeostasis cause neurodegeneration in *C. elegans* Parkinson’s models

**DOI:** 10.1038/s41419-018-0619-5

**Published:** 2018-05-10

**Authors:** Hanna Kim, Rylee J. Perentis, Guy A. Caldwell, Kim A. Caldwell

**Affiliations:** 1 Department of Biological Sciences, The University of Alabama, Tuscaloosa, AL 35487 USA; 2 Departments of Neurobiology, Neurology and Center for Neurodegeneration and Experimental Therapeutics, University of Alabama at Birmingham, Birmingham, AL 35294 USA

## Abstract

Parkinson’s disease (PD) is a complex multifactorial disorder where environmental factors interact with genetic susceptibility. Accumulating evidence suggests that mitochondria have a central role in the progression of neurodegeneration in sporadic and/or genetic forms of PD. We previously reported that exposure to a secondary metabolite from the soil bacterium, *Streptomyces venezuelae*, results in age- and dose-dependent dopaminergic (DA) neurodegeneration in *Caenorhabditis elegans* and human SH-SY5Y neurons. Initial characterization of this environmental factor indicated that neurodegeneration occurs through a combination of oxidative stress, mitochondrial complex I impairment, and proteostatic disruption. Here we present extended evidence to elucidate the interaction between this bacterial metabolite and mitochondrial dysfunction in the development of DA neurodegeneration. We demonstrate that it causes a time-dependent increase in mitochondrial fragmentation through concomitant changes in the gene expression of mitochondrial fission and fusion components. In particular, the outer mitochondrial membrane fission and fusion genes, *drp-1* (a dynamin-related GTPase) and *fzo-1* (a mitofusin homolog), are up- and down-regulated, respectively. Additionally, *eat-3*, an inner mitochondrial membrane fusion component, an OPA1 homolog, is also down regulated. These changes are associated with a metabolite-induced decline in mitochondrial membrane potential and enhanced DA neurodegeneration that is dependent on PINK-1 function. Genetic analysis also indicates an association between the cell death pathway and *drp-1* following *S. ven* exposure. Metabolite-induced neurotoxicity can be suppressed by DA-neuron-specific RNAi knockdown of *eat-3*. AMPK activation by 5-amino-4-imidazole carboxamide riboside (AICAR) ameliorated metabolite- or PINK-1-induced neurotoxicity; however, it enhanced neurotoxicity under normal conditions. These studies underscore the critical role of mitochondrial dynamics in DA neurodegeneration. Moreover, given the largely undefined environmental components of PD etiology, these results highlight a response to an environmental factor that defines distinct mechanisms underlying a potential contributor to the progressive DA neurodegeneration observed in PD.

## Introduction

Neurons are reliant on mitochondrial function due to their high-energy demands. As a result, this organelle is dynamic and fission and fusion cycles occur regularly. Mitochondria also change size or number in response to environmental cues, which can affect cellular integrity. Mitochondrial network maintenance is primarily regulated by GTPases (Drp1, Opa1) and the mitofusins (Mfn1/Mfn2). Together, these fission and fusion gene products, located on mitochondrial membranes, regulate organelle dynamics while a fission/fusion imbalance can lead to an accumulation of ultrastructural defects and eventually cell death.

Mitochondrial dysfunction is associated with Parkinson’s disease (PD) through both genetic and environmental routes. For example, mitochondrial toxicity can occur via the PD-linked recessive mutations *PARK2* (Parkin) and *PARK6* (PINK1)^1–5^. However, only 5–10% of PD cases are linked to genetic susceptibilities^6, 7^. Thus, environmental factors alone, or in combination genetic susceptibility, are thought to increase the risk of developing PD^8, 9^.

Previously, we identified a novel environmental contributor to dopaminergic (DA) neurodegeneration from *Streptomyces venezuelae* (*S. ven*). These common soil bacteria produce a secondary metabolite that causes age- and dose-dependent DA neurodegeneration in *Caenorhabditis elegans* (*C. elegans*) and cultured human DA neurons. The neurodegenerative activity, which is amphipathic, is stable and will retain neurotoxic activity after 30 min of boiling^8^. *S. ven* metabolite also increases oxidative stress, inhibits mitochondrial complex I, and reduces ATP production^8, 10, 11^. Moreover, we discovered that the cellular response to this bacterial product is epistatically regulated by *pink-1* loss-of function, suggesting that it causes DA neuronal cell death through mitotoxicity^11^.

Here we report that *S. ven* metabolite exposure is associated with an increase in mitochondrial fragmentation. There are also concomitant gene expression changes whereby *drp-1* gene expression is increased following exposure to the metabolite while *fzo-1* and *eat-3* gene expression (*C. elegans* mitofusin and OPA1, respectively) are decreased. Bacterial metabolite exposure is also associated with DA neurodegeneration through increased *pink-1-*dependent *drp-1* fission activity and *eat-3* resistance to *pink-1* mutant- and metabolite-induced neurotoxicity. Genes within the cell death pathway also interact with *drp-1* following metabolite exposure. These results demonstrate the critical response mitochondria exhibit when challenged with an environmental stressor, leading to neurodegeneration, and provide mechanistic details underlying susceptibility to neuronal cell death.

## Results

### *S. venezuelae* metabolite exposure causes mitochondrial fragmentation

As previously described^8^, *S. ven* is grown in liquid media, and then the small secondary product (MW < 300) is separated through column chromatography and extracted with dichloromethane. The *S. ven* extract fraction is collected through rotary evaporation to remove the solvent. The fraction is resuspended in ethyl acetate (EtAc) for experimentation. Hereafter, this compound will be referred to as the metabolite. EtAc is used as a negative solvent control in all experiments and does not cause any significant neurodegeneration^8, 10, 11^.

**Fig. 1:**
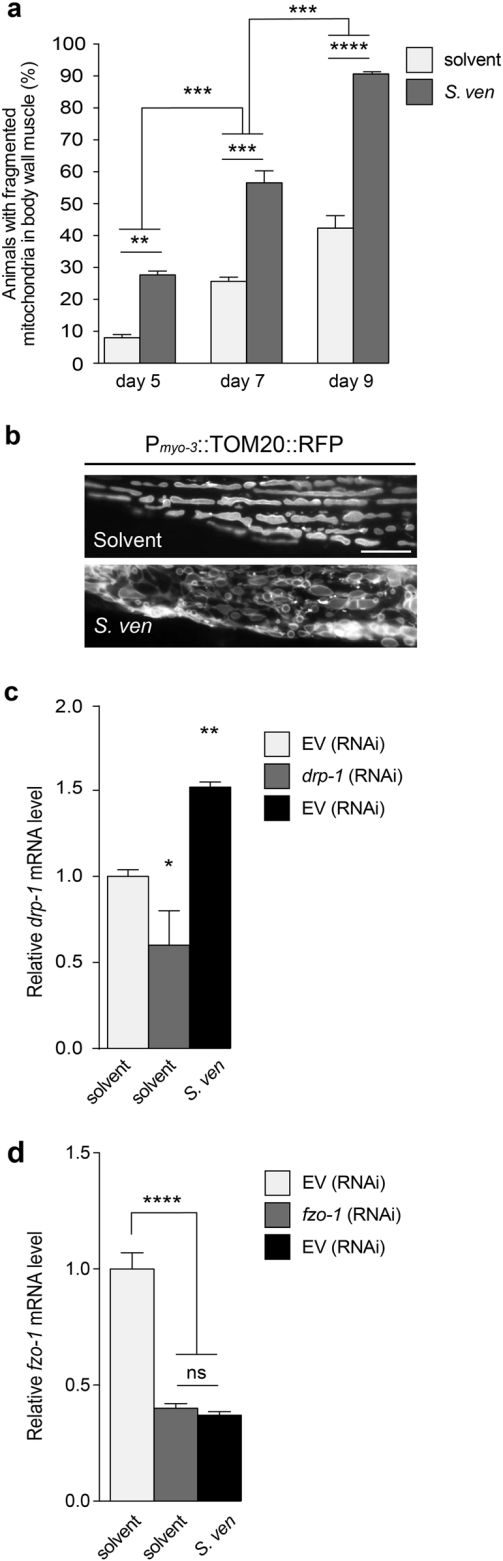
The *S. venezuelae* (*S. ven*) metabolite causes perturbations of mitochondrial fission and fusion, resulting in time-dependent mitochondrial fragmentation in *C. elegans*. **a** Quantification of mitochondrial fragmentation as indicated at young adult (5-day old), middle (7-day old), and older aged (9-day old) nematodes in response to metabolite exposure using P_*myo-3*_:TOM20:mRFP. *S. ven* metabolite significantly enhanced mitochondrial fragmentation in aging nematodes. Data represented as mean ± S.E.M.; *n* = 30 animals per replicate, three independent replicates; two-way ANOVA with Tukey’s post hoc test for multiple comparisons. **P *<* 0.01, ***P *<* 0.001, and ****P *<* 0.0001. **b** TOM20::mRFP images of the representative mitochondrial morphology following exposure of solvent or metabolite at day 5 post-hatching. The metabolite causes disordered and small donut-shaped morphology in the mitochondrial outer membrane. The scale bar is 20 *μ*m. **c**, **d** Animals exposed to solvent (EtAc) or *S. ven* metabolite were assessed for *drp-1* and *fzo-1* mRNA levels by qRT-PCR. Metabolite exposure significantly increased *drp-1* expression and reduced *fzo-1* expression levels compared to empty vector (EV) solvent control. Relative mRNA expression levels were normalized to EV control. Data represented as mean ± S.E.M.; three replicates comprising at least 100 animals each; one-way ANOVA with Tukey’s post hoc test for multiple comparisons. *P *<* 0.05, **P *<* 0.01, and ****P *<* 0.0001.

Mitochondrial toxicants often cause an imbalance between mitochondrial fission and fusion^9^. We therefore wanted to determine if *S. ven* exposure could also disrupt mitochondrial fission/fusion. We initially conducted a time course exposure with 5-, 7- and 9-day old animals. Because the body-wall muscle cells of *C. elegans* contain numerous mitochondria, it is an ideal system to investigate mitochondrial morphology. Using a reporter strain, we expressed a mitochondrial outer membrane (OMM)protein-fused to mRFP (P_*myo-3*_::TOM20::mRFP) and observed that metabolite addition significantly enhanced mitochondrial fragmentation. This was characterized by disordered and small circular-shaped mitochondria compared to solvent control at days 5, 7, and 9 post-hatching (Fig. 1a, b). We noted an increase of mitochondrial fragmentation in aging nematodes from either solvent or *S. ven* treatment; however, morphological abnormalities were significantly accelerated in metabolite-treated animals compared to solvent controls (Fig. 1a), indicating that mitochondrial morphology changes occur following metabolite exposure in a time-dependent manner.

Mitochondrial morphology reflects a balance between mitochondrial fission and fusion^7, 12, 13^. Fission removes damaged components through the OMM dynamin-related GTPase, Drp1 (*C. elegans* DRP-1) while fusion, via the conserved GTPases, Mfn1/Mfn2 (*C. elegans* FZO-1), compensates by forming long and interconnected networks. Because mitochondrial fission and fusion are primarily mediated by DRP-1 and FZO-1, respectively on the OMM, in response to stressors^14–16^, mRNA expression of *drp-1* and *fzo-1* was examined by qRT-PCR following exposure of the metabolite. Metabolite exposure significantly increased the expression levels of *drp-1* mRNA and reduced *fzo-1* expression (Fig. 1c,d). RNAi knockdown of *drp-1* or *fzo-1* served as a control. Consistent with previous observations in human cells that mitochondrial oxidative stress causes mitochondrial fragmentation through an imbalance in Drp1 and Mfn2^17^, we concluded that metabolite exposure induces a similar imbalance of fission and fusion through changes in *drp-1* and *fzo-1* gene expression in the OMM, resulting in mitochondrial fragmentation.

**Fig. 2:**
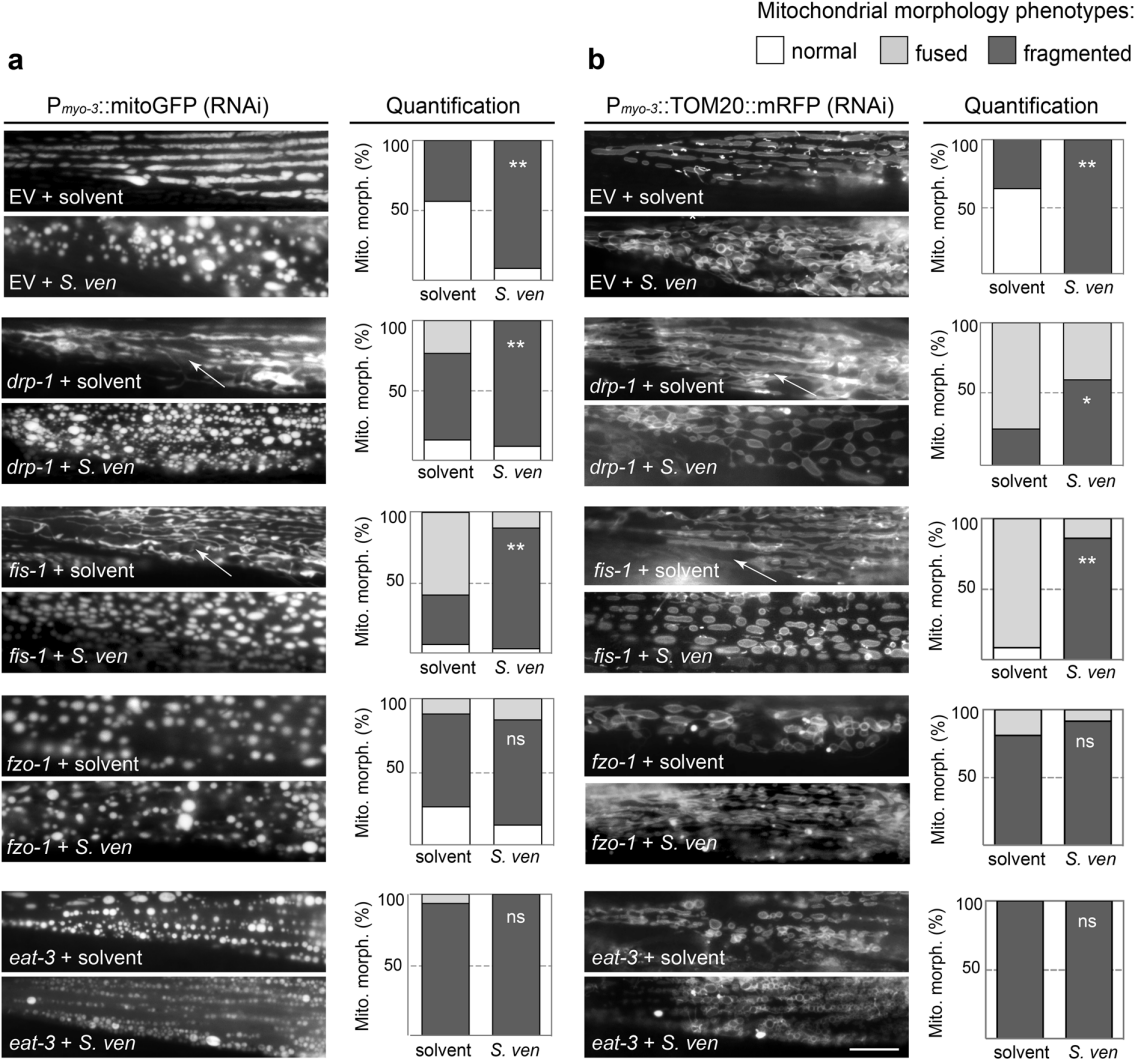
The effect of *S. ven* metabolite-mediated fission and fusion on the mitochondrial matrix (**a**) and mitochondrial outer membrane (**b**) in *C. elegans* body-wall muscles. Transgenic nematodes expressing P_*myo-3*_::mitoGFP were analyzed to monitor mitochondrial matrix in response to each treatment, and mitochondrial outer membrane was detected with P_*myo-3*_::TOM20::mRFP strain. Representative mitoGFP and TOM20 images (left panel in **a** and **b**) and quantification of the different mitochondrial morphologies observed (right panel with graphical stacks in **a** and **b**, *Y* axis represents distribution of mitochondrial morphology phenotypes in worm populations in percentage) are shown. Compared with solvent treatment, populations of RNAi-treated fission gene-associated *drp-1* and *fis-1* animals exhibited significantly more mitochondrial fragmentation following metabolite exposure, characterized by disordered and small circularly shaped mitochondria at day 8 post-hatching. Animals reduced for the fusion regulators, *fzo-1* and *eat-3*, displayed mitochondrial fragmentation following exposure to metabolite in a manner similar to solvent-treated animals, respectively. Arrows indicate representative areas of mitochondrial fusion. These data are presented as mean ± S.E.M.; *n* = 30 animals per replicate, two independent replicates; one-way ANOVA with Tukey’s post hoc test for multiple comparisons. *P *<* 0.05, **P *<* 0.01. The scale bar is 20 μm.

We quantitated morphological changes using two different mitochondrial marker strains; P_*myo-3*_::mitoGFP was used to visualize the mitochondrial matrix and P_*myo-3*_:: TOM20::mRFP for the OMM in body-wall muscle cells. In combination with RNAi depletion of OMM and inner mitochondrial membrane (IMM) fission/fusion components, these transgenic animals were used to investigate epistatic relationships. In both transgenic lines, more than half the muscle cells displayed normal tubular structures when exposed to solvent control in the EV RNAi cells (Fig. 2a, b). In notable contrast, exposure to *S. ven* metabolite resulted in a significant shift to a distinctive, circular-shaped morphology characteristic of mitochondrial fragmentation (Fig. 2a, b). RNAi knockdown of the OMM fission gene product *drp-1* increased rates of fusion (interconnected and tubular) compared to EV RNAi (Fig. 2a, b). Following metabolite exposure, a significant shift back to mitochondrial fragmentation was observed, indicating an epistatic interaction with the metabolite. Knockdown of *fis-1*, which has been shown to colocalize with DRP1 at the OMM^18^, also caused significant fusion; this phenotype could be reverted back to fragmentation with the addition of metabolite (Fig. 2a, b). RNAi knockdown of the fusion gene products *fzo-1* (OMM) and *eat-3* (IMM) resulted in a significant enhancement in mitochondrial fragmentation compared to EV control. Following treatment with metabolite, we did not observe an additional increase in fragmentation because fragmentation was already at maximum (Fig. 2a, b).

### Metabolite-mediated effects on mitochondrial function

**Fig. 3:**
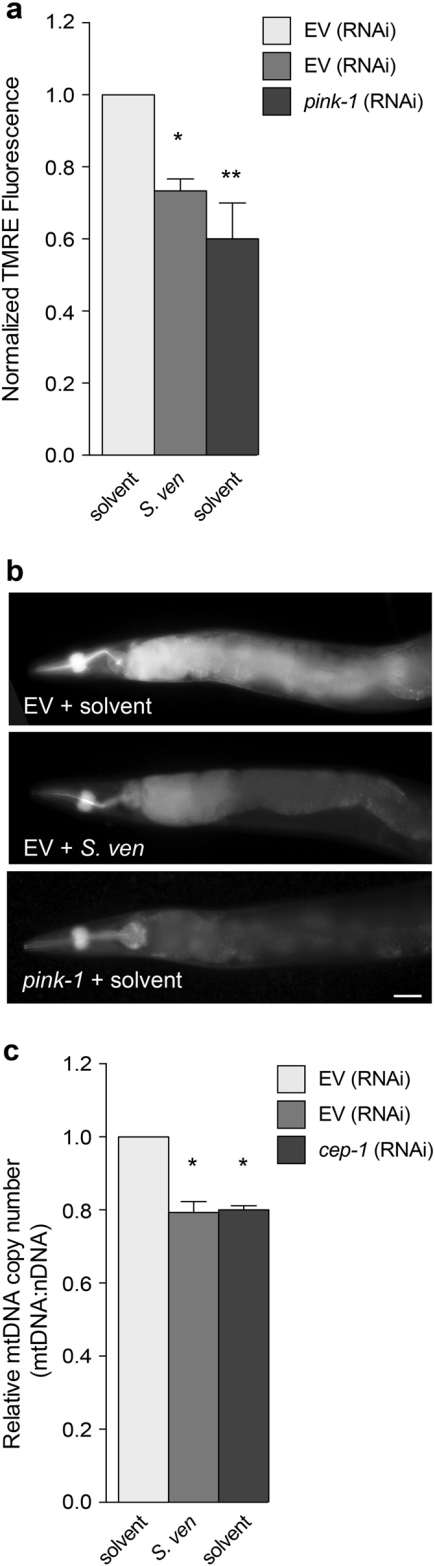
The effect of *S. ven* metabolite-mediated fission and fusion on mitochondrial function. **a** Relative mitochondrial uptake of the fluorescent dye tetramethylrhodamine ethyl ester (TMRE) was assessed for the mitochondrial membrane potential (ΔΨ_m_) of *C. elegans*. Animals (wild-type N2) exposed to *S. venezuelae* metabolite had a significantly lower ΔΨ_m_ then solvent-treated EV control animals at day 8 post-hatching. RNAi knockdown of *pink-1* was used as a positive control. Relative TMRE fluorescent intensity was normalized to EV solvent control. These data are presented as mean ± S.E.M.; *n* = 30 animals per replicate, three independent replicates; one-way ANOVA with Dunnett’s post hoc test for multiple comparisons. *P *<* 0.05, **P *<* 0.01. **b** Representative images of TMRE-stained nematodes following treatment with solvent and metabolite, respectively. The scale bar is 5 *μ*m. **c** Quantitation of mitochondrial DNA (mtDNA) copy number by qRT-PCR in N2 animals. The metabolite caused a relative decrease of mtDNA copy number in comparison to EV solvent control. *cep-1* (RNAi) was included as a positive control. Data represented as mean ± S.E.M.; three biological and three technical replicates; one-way ANOVA with Tukey’s post hoc test for multiple comparisons. *P *<* 0.05

Abnormal mitochondrial fission/fusion is associated with mitochondrial membrane potential (ΔΨ_m_) collapse^15, 19^. To explore the hypothesis that the metabolite alters ΔΨ_m_, we measured the relative mitochondrial uptake of the fluorescent dye tetramethylrhodamine ethyl ester (TMRE) in live nematodes following *S. ven* exposure. This lipophilic cation dye has been used previously in *C. elegans* as an indicator of mitochondrial activity^20–22^. We used RNAi knockdown of the familial recessive PD gene, *pink-1*, as a positive control. Animals exposed to metabolite or *pink-1* (RNAi) displayed significant decreases in TMRE fluorescence in comparison with solvent-treated EV control group at day 8 (Fig. 3a, b).

Given the observed perturbations of fission/fusion by metabolite, it is possible that mitochondrial DNA (mtDNA) becomes unstable and accumulates mutations as a consequence of defective mitochondrial fusion. Thus, we assessed mtDNA levels in response to the metabolite. Because mtDNA is more sensitive than nuclear DNA (nDNA) to environmental stress exposures^23^, the mtDNA:nDNA ratio was determined in N2 wild-type worms treated with metabolite by qRT-PCR. As a positive control, we depleted the *C. elegans* homolog of p53 (*cep-1*) by RNAi, which causes sensitivity to mtDNA damage as previously described^24^. Compared to solvent treatment, metabolite-treated animals displayed a significant decrease in the mtDNA:nDNA ratio in comparison with solvent-only animals (Fig. 3c). Taken together, these results suggest that a metabolite-induced imbalance of fission/fusion exacerbates mitochondrial toxicity.

### Down regulation of *eat-3* suppresses neurotoxicity caused by the metabolite

**Fig. 4:**
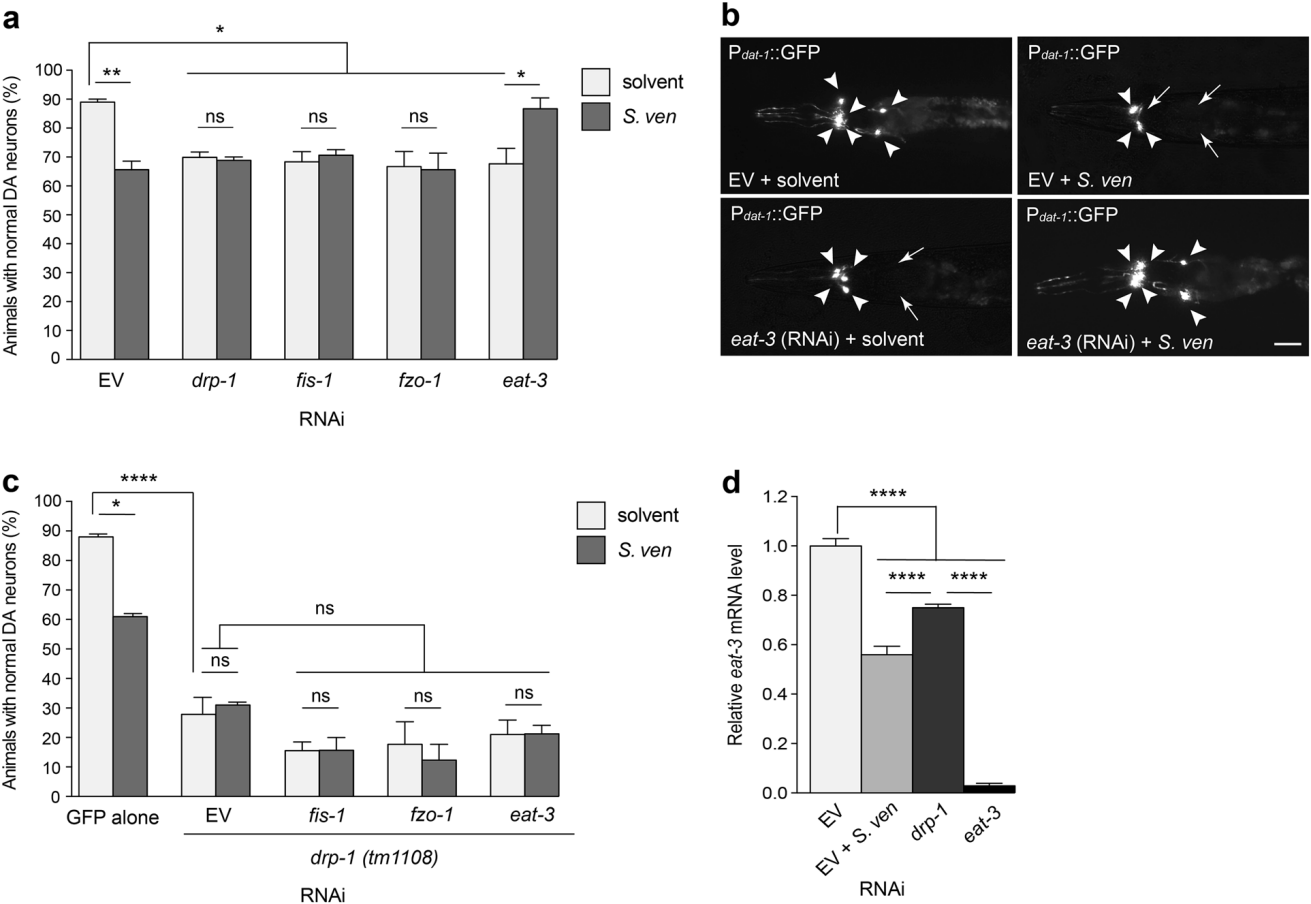
Down-regulation of *eat-3* suppresses neurotoxicity caused by the metabolite. **a**, **b** RNAi knockdown of fission (*drp-1* and *fis-1*) and fusion (*fzo-1* and *eat-3*) components with solvent caused DA neurodegeneration at day 9 post-hatching. With metabolite exposure, RNAi treated with *drp-1*, *fis-1*, or *fzo-1* animals did not exhibit enhanced DA neurotoxicity compared to the corresponding solvent control. In contrast, a combination of *S. ven* metabolite and RNAi targeting *eat-3* resulted in significant resistance to neurotoxicity. The scale bar is 10 μm. These data are presented as mean ± S.E.M.; *n* = 30 animals per replicate, three independent replicates; one-way ANOVA with Tukey’s post hoc test for multiple comparisons. *P *<* 0.05, **P *<* 0.01. Representative images of the six anterior dopaminergic neurons are shown in (**b**), with degenerating or missing neurons marked by arrows and normal neurons indicated by arrowheads. **c** An RNAi-sensitive strain with GFP expression in DA neurons was crossed to *drp-1(tm1108)* mutant animals. Homozygosity was confirmed by PCR. Loss-of-function *drp-1(tm1108)* exacerbated DA neurotoxicity compared with GFP only solvent control at day 9 post-hatching. RNAi knockdown of *eat-3* did not result in resistance of neurotoxicity as observed in Fig. 4a. Data are represented as mean ± S.E.M; *n* = 30 animals per replicate, three independent replicates; one-way ANOVA with Tukey’s post hoc test for multiple comparisons. *P < 0.05, *P < 0.0001. **d** *eat-3* mRNA levels were measured by qRT-PCR in animals treated with solvent (EtAc), *S. ven* metabolite, *drp-1* (RNAi), or *eat-3* (RNAi). The animals treated with metabolite showed significant lower *eat-3* mRNA levels than when *drp-1* was depleted by RNAi. Data represented as mean ± S.E.M.; three replicates comprises at least 100 animals each; one-way ANOVA with Tukey’s post hoc test for multiple comparisons. ****P *<* 0.0001.

Considering the severe mitochondrial morphological changes induced by *S. ven* exposure, we sought to evaluate how modulating fission/fusion impacts DA neurodegeneration. Our analysis was performed through RNAi knockdown of mitochondrial fission/fusion components following exposure of metabolite in an RNAi strain that selectively enables RNAi only in DA neurons^25, 26^. Consistent with the effect on mitochondrial morphology observed in body-wall muscle cells, RNAi depletion of fission (*drp-1* and *fis-1*) and fusion (*fzo-1* and *eat-3*) genes resulted in significant DA neurodegeneration in comparison with solvent-only EV control at day 9 post-hatching (Fig. 4a). When metabolite was added, it enhanced degeneration in EV RNAi conditions, but it did not result in further degenerative changes in *drp-1*, *fis-1* or *fzo-1* RNAi knockdown conditions (Fig. 4a, b). However, a combination of *eat-3* (RNAi) and metabolite exposure resulted in resistance to DA neurotoxicity (Fig. 4a, b). A previously reported study suggested that the OMM fission component *drp-1* acts upstream of *eat-3*, an IMM fusion component, and that mutual compensation occurs for physiological defects^27^. Since we observed that metabolite increases *drp-1* gene expression levels (Fig. 1c), it is possible that depletion of *eat-3* could be compensated by metabolite-induced *drp-1*, or vice versa, providing resistance to DA neurodegeneration. To explain the interaction between *eat-3* and metabolite-induced *drp-1*, we examined the effect of *eat-3* (RNAi) on DA neurodegeneration in a *drp-1(tm1108)* null mutant background crossed to the DA neuron-sensitive RNAi strain. We hypothesized that if metabolite-induced *drp-1* interacts with *eat-3* (RNAi), then it would not exhibit the resistance phenotype under the *drp-1* null mutant background. Because mitochondrial fission/fusion components are interdependent, we also knocked down *fis-1* and *fzo-1* in the *drp-1* mutant background. RNAi depletion of *fis-1* or *fzo-1* resulted in severe neurodegeneration in the *drp-1* mutant background; solvent and the addition of metabolite did not cause further neurodegeneration (Fig. 4c). This indicated that *drp-1* acts as an upstream component for fission/fusion as it pertains to DA neurodegeneration. Interestingly, a combination of metabolite and *eat-3* (RNAi) in the *drp-1* null background no longer displayed neuroprotection against the metabolite, indicating that there is epistatic regulation between *eat-3* depletion and metabolite-induced *drp-1* activity (Fig. 4a vs. c). We also monitored *eat-3* transcriptional activity by qRT-PCR to determine the interaction between *drp-1* and *eat-3*. When *drp-1* was depleted by RNAi, the *eat-3* gene expression levels were significantly higher than when *drp-1* was activated by metabolite treatment (Fig. 4d). These results provide evidence that response to metabolite involves *drp-1* fission activity, which genetically interacts with *eat-3* in a compensatory manner.

### DRP-1-dependent metabolite induction of the cell death pathway

**Fig. 5:**
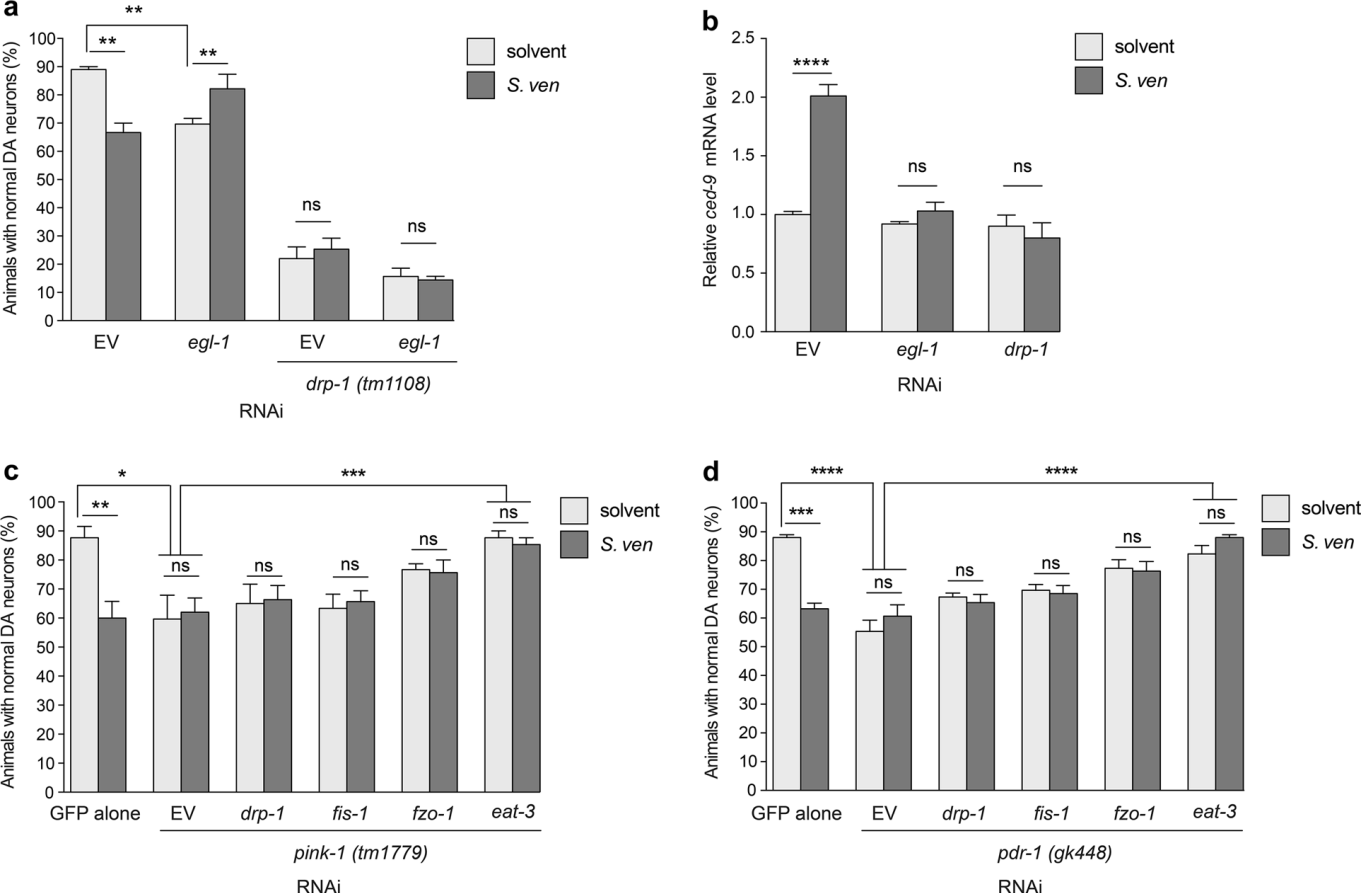
Exposure to *S. ven* metabolite is associated with cell death. **a** Reduction of *egl-1* (RNAi) caused neurodegeneration in comparison with EV solvent control; however, addition of metabolite showed significant reduction of neurotoxicity. Under the *drp-1(tm1108)* mutant background, the animals exhibited severe neurodegeneration, and the addition of metabolite did not cause further neurodegeneration in EV solvent control or *egl-1* RNAi. These data are presented as mean ± S.E.M.; *n* = 30 animals per replicate, three independent replicates; one-way ANOVA with Tukey’s post hoc test for multiple comparisons. **P *<* 0.01. **b** *ced-9* mRNA levels were measured by qRT-PCR. Metabolite exposure resulted in a significant increase in *ced-9* transcriptional activity compared to EV solvent control, however, there was no significant change in *ced-9* expression level when *egl-1* or *drp-1* was depleted (RNAi). Data are represented as mean ± S.E.M; three replicates comprised of at least 100 animals each; one-way ANOVA with Tukey’s post hoc test for multiple comparisons. ****P *<* 0.0001. **c**, **d** A dopaminergic (DA) neuron RNAi-sensitive strain with GFP expression in DA neurons was crossed to strains harboring mutant alleles for *pink-1(tm1779) or pdr-1(gk448)*. Homozygosity was confirmed by PCR. Loss of either *pink-1(tm1779)* (**c**) or *pdr-1(gk448)* (**d**) function enhanced DA neurodegeneration with or without metabolite compared to GFP only solvent control at day 9 post-hatching. RNAi knockdown of *eat-3* resulted in significant resistance to both *pink-1* (**c**) and *pdr-1*-induced (**d**) neurotoxicity. Data presented as mean ± S.E.M.; *n* = 30 animals per replicate, three independent replicates; one-way ANOVA with Tukey’s post hoc test for multiple comparisons. *P *<* 0.05, **P < 0.01, ***P < 0.001, ****P < 0.0001.

To explore neurodegeneration within the context mitochondrial fission we turned to CED-9, a protein involved in both proapoptotic and antiapoptotic activities in *C. elegans*. More specifically, CED-9, an ortholog of BCL2-like protein, has been reported to interact with DRP-1 to promote mitochondrial fission^28^. Since RNAi knockdown of CED-9 led to lethality, we examined EGL-1, an upstream activator of the cell death pathway, which is specifically required to regulate CED-9^28, 29^. RNAi knockdown *egl-1* enhanced DA neurodegeneration in comparison with EV solvent control; however, metabolite addition significantly attenuated neurotoxicity (Fig. 5a). We also introduced the *drp-1(tm1108)* mutant background, which caused severe neurotoxicity; here, the addition of metabolite did not attenuate neurodegeneration in *egl-1* RNAi (Fig. 5a). We then examined *ced-9* expression level by qRT-PCR (Fig. 5b). We observed that it was significantly increased following *S. ven* exposure, however, under *egl-1* or *drp-1* RNAi depletion there was no significant change of *ced-9* expression with *S. ven* (Fig. 5b). These data suggest a possible interaction between CED-9 and DRP-1 following metabolite induction.

### Metabolite induces PINK-1/PDR-1-dependent fission

Mitochondrial fission is also associated with the PINK1/Parkin pathway^30, 31^. These proteins generally function together to regulate mitochondrial homeostasis^32–34^ in part by promoting DRP1-dependent mitochondrial fission^30, 31^. We hypothesized that if metabolite-induced fission is independent of PINK-1, it would suppress the *pink-1* mutant phenotype. Conversely, if dependent on PINK-1 function, metabolite addition would not enhance neurodegeneration when *pink-1* was depleted. We assessed these predictions in null mutant alleles of *pink-1(tm1779)* and *pdr-1(gk448)* (corresponding to human PINK1 and Parkin, respectively), which were crossed into the DA-neuron-specific RNAi strain. Consistent with prior reports^32, 35^, loss of *pink-1* enhanced DA neurodegeneration compared to GFP only animals (solvent comparison) (Fig. 5c). Metabolite addition did not enhance neurotoxicity from *pink-1* loss (Fig. 5c). RNAi knockdown of *drp-1* in the *pink-1* loss-of-function background did not further enhance DA neurodegeneration, indicating that metabolite-induced *pink-1* neurodegeneration is dependent on DRP-1 fission. We also found that reduction of *eat-3* (RNAi) significantly suppressed *pink-1*-induced neurodegeneration with or without metabolite exposure (Fig. 5c). Similar results were obtained under the *pdr-1(gk448)* mutant background (Fig. 5d). These data suggest that down regulation of *eat-3* is neuroprotective in *pink-1/pdr-1* mutant conditions.

### AICAR-mediated AMPK activation rescues metabolite and *pink-1*-induced dopaminergic neurodegeneration

**Fig. 6:**
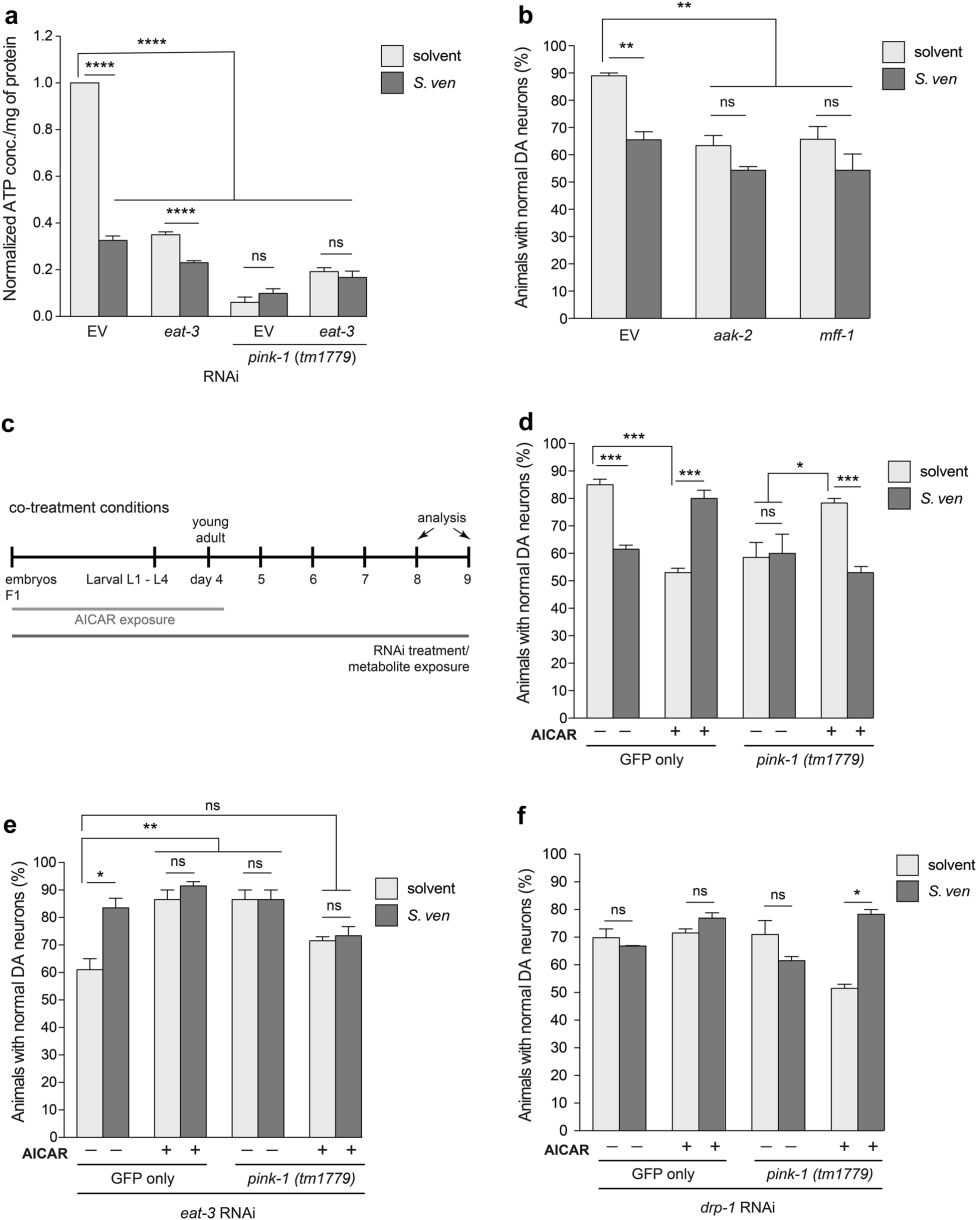
AICAR-mediated AMPK activation rescues metabolite-induced dopaminergic neurodegeneration. **a** The effect of *eat-3* RNAi on ATP levels in *pink-1(tm1779)* mutant animals after exposure of solvent (EtAc) or metabolite. ATP content was measured in young adult nematodes using a luciferase-based assay. Values are mean ± S.E.M.; *n* = 3 independent samples with 100 worms in each; one-way ANOVA with Tukey’s post hoc test for multiple comparisons. ****P *<* 0.0001. **b** Reduction of either *aak-2* or *mff-1* enhanced DA neurotoxicity with or without metabolite in comparison with EV solvent control. Data represented as mean ± S.E.M.; *n* = 30 animals per replicate, three independent replicates; one-way ANOVA with Tukey’s post hoc test for multiple comparisons. **P *<* 0.005. **c** A timeline representing an experimental paradigm depicting the relative length of *S. ven* metabolite exposure and AICAR treatment. The abbreviations L1–L4 are the larval stages of *C. elegans*, and the ‘young adult’ designations represent days post-hatching. F1 animals were treated with either RNAi and/or *S. ven* metabolite from hatching to the day of analysis; neurodegeneration assays were performed at day 9. Animals were treated with 1 mM AICAR from hatching until day 4. **d** Animals expressing only GFP in the DA neurons treated with AICAR (alone) displayed enhanced DA neurodegeneration compared to solvent controls; however, metabolite-induced neurotoxicity was reduced by AICAR treatment. Furthermore, AICAR rescued neurotoxicity caused by *pink-1(tm1779)* in comparison to non-AICAR treated (solvent) animals with *pink-1* mutation. AICAR did not rescue DA neuron cell death from the combined stress of metabolite and *pink-1*. Data are presented as mean ± S.E.M.; *n* = 30 animals per replicate, three independent replicates; one-way ANOVA with Tukey’s post hoc test for multiple comparisons. *P *<* 0.05, ***P *<* 0.001. **e** AICAR-mediated AMPK activation was sufficient to protect neurodegeneration caused by *eat-3* deficiency (RNAi) alone or with metabolite, however, it could not reduce neurotoxicity in the absence of *pink-1* function *(tm1779)* and *eat-3* RNAi together. Data represented as mean ± S.E.M.; *n* = 30 animals per replicate, three independent replicates; one-way ANOVA with Tukey’s post hoc test for multiple comparisons. *P *<* 0.05, **P *<* 0.01. **f** AICAR-mediated AMPK activation failed to attenuate neurotoxicity from *drp-1* (RNAi) depletion in the GFP only or *pink-1(tm1779)* mutant background; however, AICAR suppressed neurotoxicity in the *pink-1* mutant background in combination with metabolite exposure. These data are presented as mean ± S.E.M.; *n* = 30 animals per replicate, three independent replicates; one-way ANOVA with Tukey’s post hoc test for multiple comparisons. *P *<* 0.05.

We previously reported that the metabolite can decrease ATP production^10^. Since neurotoxicity from either metabolite and/or the *pink-1* mutant was reduced by *eat-3* depletion, we next wanted to determine whether down regulation of *eat-3* acts as a compensatory mechanism to maintain adequate ATP levels. We performed an ex vivo ATP assay on *pink-1(tm1779)* mutant nematodes treated with *eat-3* RNAi with metabolite. ATP levels were significantly decreased in metabolite-treated control animals (EV), *pink-1* mutant and *eat-3* RNAi knockdown animals (Fig. 6a). However, down regulation of *eat-3* did not exhibit an effect on ATP levels (Fig. 6a).

AMP-activated protein kinase (AMPK) is activated by ATP depletion from mitochondrial stress and enhances mitochondrial biogenesis to supplant energy deficiency^16, 36^. Moreover, AMPK is neuroprotective against the mitochondria complex I inhibitors 1-methyl-4-phenylpyridinium (MPP^+^) and rotenone^36, 37^. Therefore, we investigated whether depletion of the *C. elegans* homolog of AMPK, *aak-2*, and an AMPK substrate and DRP-1 receptor, MFF-1, caused DA neurodegeneration in response to metabolite. Knockdown of both gene products resulted in significant DA neurodegeneration while the addition of metabolite did not further enhance neurodegeneration, indicating this process is modulated by the metabolite (Fig. 6b). We next examined the activation of AMPK by a pharmacological method to determine if we could ameliorate metabolite-induced neurotoxicity. Animals were treated with 5-aminoimidazole-4-carboxamide ribonucleotide (AICAR), an activator of AMPK (Fig. 6c), and analyzed for DA neurodegeneration. AICAR treatment caused significant DA neurodegeneration under normal conditions (GFP only with solvent). Interestingly, AICAR attenuated neurotoxicity when animals were exposed to metabolite (Fig. 6d). We further examined whether AMPK activation would suppress neurodegeneration from *pink-1* loss-of-function. Compared with non-AICAR treated *pink-1(tm1779)* animals, AICAR treatment was significantly neuroprotective in solvent-only animals (Fig. 6d). However, AICAR could not attenuate neurotoxicity from the combination of *pink-1* loss-of-function and metabolite (Fig. 6d).

We further delved into the effect of AMPK activation under RNAi knockdown of *eat-3* or *drp-1* (Fig. 6e,f, respectively). We observed that AICAR-mediated AMPK activation was sufficient to protect against *eat-3* deficiency (GFP only) but it failed to suppress neurotoxicity in the *pink-1* mutant background (Fig. 6e). Conversely, AICAR-mediated AMPK activation did not protect against *drp-1* deficiency in the GFP only background, but it was able to suppress neurotoxicity in the *pink-1* mutant background in combination with the metabolite (Fig. 6f).

## Discussion

Perturbations in mitochondrial fission/fusion have been reported as critical mechanisms during neurodegeneration^33, 38, 39^. Although genetic risk factors are associated with this process, the interaction between an environmental contributor and mitochondrial dynamics remains poorly understood. In an effort to uncover potentially more prevalent sources of environmental influence on neurodegeneration, we previously identified a metabolite from the common soil bacterium, *S. venezuelae*, that induces DA neuron cell death by targeting mitochondria^8, 10, 11^. Here, we extend these studies by examining mitochondrial dynamics and mechanistically discerning genetic modulators altering its neurotoxicity in vivo.

We initially determined that the metabolite enhances mitochondrial fragmentation in a time-dependent manner (Fig. 1a). We were curious to know if the mitotoxic properties of *S. ven* could be distinguished from other mitochondrial complex I inhibitors. This was performed by thoroughly examining mitochondrial fission/fusion changes following metabolite exposure in combination with RNAi knockdown of mitochondrial fission or fusion gene products in *C. elegans* body-wall muscle cells and DA neurons. We discovered that the mechanism of action underlying *S. ven* neurotoxicity is distinct from that of either rotenone or MPP^+^.

*S. ven* exposure increases mitochondrial fragmentation (Figs. 1, 2) while rotenone decreases fission and induces fusion^40–42^. Likewise, *drp-1* gene expression increases following *S. ven* treatment (Fig. 1c) but it decreases following rotenone treatment^41^. Similar to *S. ven*, MPP^+^ increases mitochondrial fragmentation and increases DRP1 levels^43, 44^. However, genetic inactivation of *drp-1* in SH-SY5Y cells blocks MPP^+^ mitochondrial fragmentation^43^ whereas fragmentation still occurs in *C. elegans* when *drp-1* is knocked down following treatment with *S. ven* (Fig. 2). While all three of these neurotoxic substances inhibit mitochondrial complex I, their mitotoxic mechanisms of action are clearly distinguishable.

We have previously reported that the metabolite upregulates two pathways associated with mitochondrial dysfunction, the mitochondrial unfolded protein response pathway (UPR^mt^) and intracellular ROS accumulation^10^. The UPR^mt^ can be activated by a mitonuclear protein imbalance that shifts the relative nDNA and mtDNA levels^45^. Following metabolite exposure, a mtDNA:nDNA imbalance was identified (Fig. 3c) and we have shown that the UPR^mt^ was upregulated in response to metabolite^10^. The UPR^mt^ signaling pathway regulates > 900 gene products including nDNA-encoded mitochondrial molecular chaperones, the mitochondrial protease CLPP, and DRP-1^46^.

While ROS can cause toxicity and alter mitochondrial function at higher levels, it has also been shown, at lower levels, to be beneficial to *C. elegans* where it extends lifespan^47^. Likewise, lifespan extension has been also noted with reduction of mitochondrial electron transport chain components^48^. Such differences are interesting and may reflect tissue-specific distinctions in exposure to acute and chronic stressors. ROS overproduction both enhances DRP-1 and reduces FZO*-*1 activities, resulting in mitochondrial fragmentation;^17^ likewise, *S. ven* increases *drp-1* gene expression and decreases *fzo-1* gene expression (Fig. 1).

The impact of increased *drp-1* gene expression following *S. ven* exposure was examined in more detail in our study. Notably, *eat-3* RNAi attenuated metabolite-induced neurodegeneration, while other combinations of *S. ven* and fission/fusion genes did not alter levels of neurodegeneration. We hypothesized that, in the presence of metabolite-induced stress, the absence of *eat-3* led to an increase in mitochondrial fission. Indeed, through an epistasis analysis we determined that *eat-3* was no longer neuroprotective against *S. ven* in the *drp-1* null mutant background (Fig. 4c), indicating a genetic interaction between *drp-1* and *eat-3*. The interaction between *drp-1* and *eat-3* was also examined by monitoring *eat-3* transcriptional activity. When *drp-1* was depleted by RNAi, *eat-3* levels were significantly higher than when *drp-1* was activated by metabolite (Fig. 4d). These results provide evidence that the metabolite induces *drp-1*-mediated fission activity, which genetically interacts with *eat-3* in a compensatory manner.

**Fig. 7:**
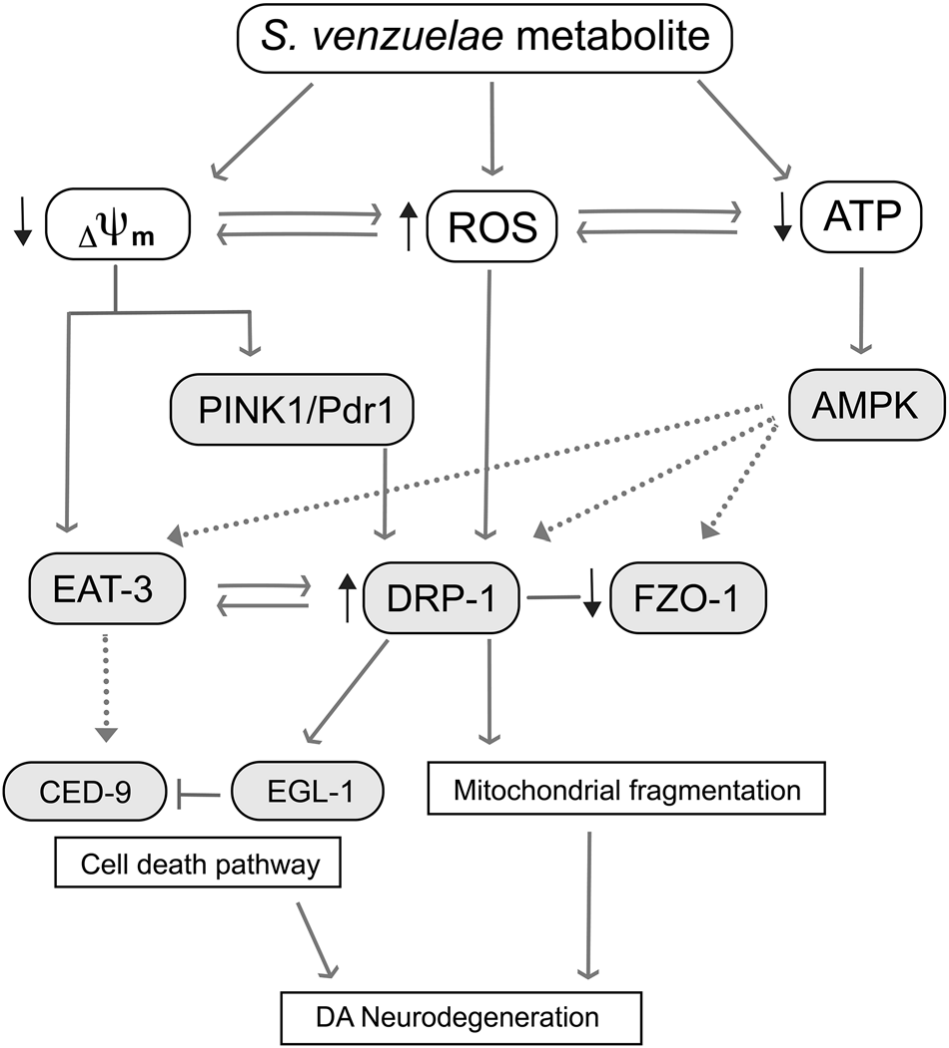
Proposed mechanism of altered mitochondrial homeostasis caused by *S*. *ven* metabolite-induced toxicity. This experimental model illustrates our current understanding of the effect of *S. ven* metabolite on mitochondrial homeostatic mechanisms in the development of neurodegeneration. metabolite-induced ROS gradually impairs mitochondria through mitochondrial complex I damage, energy deprivation, and eventually, the loss of ΔΨ_m_. As ΔΨ_m_ is decreased, *C. elegans* PINK-1 and PDR-1 would be mobilized to the OMM, which would then recruit the OMM fission factor, DRP-1, to the OMM to degrade the damaged organelle, resulting in mitochondrial fragmentation. Moreover, proteolytic cleavage of EAT-3 by the decline in ΔΨ_m_ and increased DRP-1 could lead to neuron cell death. In our genetic studies, the reduction of *eat-3* (RNAi) attenuated neurotoxicity induced by metabolite or in the *pink-1* mutant background, demonstrating that *eat-3* depletion (RNAi) might counteract cell death and have a role as an antiapoptotic factor when the cell death pathway is activated by DRP-1. Moreover, we observed a genetic interaction between *drp-1* and *egl-1*, a component of the cell death pathway as well as increased mRNA expression of *ced-9* in the presence of metabolite. Another DRP-1-related response observed involves *C. elegans* AAK-2 (AMPK), which is a candidate signaling molecule and known regulator of mitochondrial biogenesis. The AMPK pathway converges with the gene expression of mitochondrial fission and fusion genes, such as *drp-1* and *eat-3*, representing a prospective mechanism of response to *S. ven* exposure.

It has been reported that low ΔΨ_m_ in mitochondria serves as a trigger for PINK-1 accumulation on OMM to signal the degradation of damaged organelles through autophagy^5, 30, 49, 50^. This process requires DRP1 fission to generate small mitochondrial particles for efficient organelle degradation^5, 30, 49, 50^. We previously reported that the metabolite-induced PINK-1-dependent autophagy^10, 11^. Here, metabolite-induced *drp-1* function is dependent on PINK-1 (Fig. 5c). Therefore, it is possible that metabolite-induced ROS could enhance *drp-1* gene expression directly and lower ΔΨ_m_ caused by metabolite exposure (Fig. 3a,b), which would also induce PINK-1-dependent DRP-1 fission for removal of damaged mitochondria through autophagy (Fig. 7).

It was previously reported that DRP-1 recruitment to mitochondria is promoted by EGL-1 and CED-9 in *C. elegans*^28, 51^. Exposure to metabolite causes an increase in *ced-9* gene expression (Fig. 5b), and neurotoxicity from disrupting *egl-1* was reduced by metabolite (Fig. 5a). This observation suggests a possible interaction between PINK-1/PDR-1 and the apoptotic pathway. PINK-1 has been shown as mediator of cytochrome c release, which is a key component for initiating caspase acitivity^52^. Collapse of ΔΨ_m_ by metabolite could trigger PINK-1 accumulation on OMM, gathering DRP-1 for fission while it induces the cell death pathway.

Previously, it was shown that an increase in DRP-1 activity occurred in response to mitochondrial dysfunction through AMPK, a key regulator of cellular energy metabolism^16, 53^. AMPK can promote mitochondrial division and is activated in response to mitochondrial complex inhibitors, such as rotenone and antimycin A^16^. AMPK suppressed the DA neurotoxicity associated with metabolite exposure in N2 wildtype, *eat-3* (RNAi), and *pink-1*; *drp-1*(RNAi) animals (Fig. 6). These data indicate that AMPK has an important role underlying the mechanism of metabolite-induced DA neurodegeneration. Although further work will be required to elucidate how the metabolite triggers mitochondrial dysfunction, modulation of AMPK activity, an established effector of mitochondrial biogenesis, is a potential means by which changes in *drp-1* and *eat-3* gene transcription levels are regulated in response to metabolite (Fig. 7).

In considering the etiological understanding of PD, there remains an urgent need to define putative gene-by-environment interactions that underlie neurodegeneration, well beyond current explanations. Our data provide evidence that a bacterial source of an environmental toxin causes DA neuron cell death through mitotoxicity and that established genetic susceptibility factors for PD, such as *pink-1* mutation, exacerbate neurodegeneration. Chronic exposure of the *S. ven* metabolite causes alterations in mitochondrial fission/fusion, resulting in neurodegeneration. This study advances our understanding of the mitochondrial homeostatic mechanism in response to an environmental stressor that induces neuronal cell death and, with the identification of AMPK as a neuroprotective agent, provides an avenue for subsequent investigation of environmental exposures and potentially targeted modulation of their impact on neurodegeneration.

## Materials and Methods

### *C. elegans* strains

Nematodes were maintained through well-established procedures^54^. The following strains were provided by the CGC: SJ4103 [*zcls14*(P_*myo-3*_::mitoGFP)], PS6192 [*syls243*(P_*myo-3*_::TOM20::mRFP + *unc-119*( + ) + pBS Sk + )], and CU6372 [*drp-1(tm1108)* IV]. Strain *pink-1(tm1779)* was provided by the Mitani laboratory through the National BioResource Project of the MEXT, Japan. Other strains included UA202 [*vtIs7*(P_*dat-1*_::GFP); *sid-1*

*(pk3321*)*; baIn33*(P_*dat-1*_::*sid-1*, P_*myo-2*_::mCherry)], UA312 [*vtIs7*(P_*dat-1*_::GFP); *drp-1(tm1108)*; *baIn33*(P_*dat-1*_::*sid-1*, P_*myo-2*_::mCherry), *sid-1(pk3321*)], UA313 [*vtIs7*(P_*dat-1*_::GFP); *pink-1(tm1779)*; *sid-1(pk3321*)*; baIn33*(P_*dat-1*_::*sid-1*, P_*myo-2*_::mCherry)], UA316 [*vtIs7*(P_*dat-1*_::GFP); *pdr-1(gk448)*; *sid-1(pk3321*)*; baIn33*(P_*dat-1*_::*sid-1*, P_*myo-2*_::mCherry)].

### Construction of *drp-1 (tm1108)*, *pink-1 (tm1779)*, and *pdr-1 (gk448)* RNAi strains

The mutant animals were crossed into strain UA202 [*sid-1(pk3321*)*; baIn33*(P_*dat-1*_::*sid-1*, P_*myo-2*_::mCherry); (P_*dat-1*_::GFP)] to generate UA312, UA313, and UA316, respectively. The *sid-1(pk3321)* point mutation was detected using the following primers: forward CTTCTTGTATACTGAACGACG and reverse GCACAGTTATCAGATTTGCA. PCR samples were digested with the restriction enzyme ApoI for 2 h at 50 °C. The products were examined by agarose gel electrophoresis. Total PCR product is 874 bp; *sid-1(pk3321)* cut by ApoI restriction enzyme generated two fragments: 523 bp and 350 bp. After crossing mutant strains containing these alleles, the molecular lesions were probed using the following primers:

*drp-1 (tm1108)* Forward: CCAGACTTCGATGCCGTG

*drp-1 (tm1108)* Reverse: CCTCCGCATAGCTCAGTT G

*pink-1(tm1779)* Forward: GTTACAAGGCGAGCCTGAAAG

*pink-1(tm1779)* Reverse: GAAGCCTCGGGCTTATTAAGG

*pdr-1(gk448)* Forward: CACTTACGCAAGTGCTTCTTCG

*pdr-1(gk448)* Reverse: GTACGTGAGTTAGAGCTGC

### Isolation and extraction of *S. venezuelae* metabolite

*S. venezuelae* metabolite was generated as previously described^10, 11^. Briefly, spores from *S. venezuelae* (ARS NRRL ISP-5230) were rehydrated at a density of 1 × 10^8^ in 5 L SYZ media and were grown at 30 °C in a shaker for 3 weeks. For harvesting samples, cell debris was removed by centrifugation at 10,000 × *g* for 10 min and supernatants were sequentially passed through eight PES filter membranes with the following range of pore sizes: 11, 6, 2.7, 1.7, 1.2, 0.7, 0.45, and 0.22 *μ*m. The conditioned media was extracted with an equal volume of dimethylchloride for 3 times using a separatory funnel. The dimethylchloride organic layers were collected, dried, and resuspended at a 1 × concentration, which is equivalent to 1 μl of metabolite resuspended in 1 ml EtAc.

### *S. venezuelae* metabolite treatment

For the assays in this study, 20 × concentration of metabolite was used [2 μl of the concentrated stock solution was reconstituted in 100 μl EtAc] or EtAc (solvent control) was added to the surface of bacterial lawn on nematode growth medium (NGM) Petri plates (60 mm diameter) and allowed to dry. Animals were transferred to freshly made plates every other day and were exposed to the metabolite or solvent from hatching until the day of analysis, which was performed with the investigator blinded to sample identities. It should be noted that 20 × is the standard concentration used when analyzing GFP-only DA neurons. When our lab introduces a proteostasis stressor such as α-synuclein to DA neurons a 5 × concentration is appropriate^11^.

### AICAR assay

AICAR (Adipogen, San Diego, CA, USA) was added directly to agar media at a final concentration of 1 mM as previously described^55^. Nematodes were exposed to AICAR from hatching to day 4, then were transferred to corresponding treatment plates without AICAR, as described in Fig. 6c. Dopaminergic neurodegeneration assays were then performed with the investigator blinded to sample identities using normal assay conditions.

### RNAi treatments

RNAi feeding constructs were obtained from the *C. elegans* Ahringer library^56^. RNAi feeding clones were inoculated in LB culture with 100 μg/ml ampicillin, and grown overnight at 37 °C, shaking. Small (35 mm diameter) NGM plates containing IPTG-ampicillin (1 mM IPTG, 100μg/ml ampicillin) were seeded with 250 μl of RNAi bacteria culture and allowed to dry. RNAi feeding clones and double-stranded RNA was induced overnight at 20 °C. *S. venezuelae* metabolite solution [2 μl (20 × ) of the concentrated stock solution is reconstituted in 100 μl EtAc] or EtAc (solvent control) alone was spread onto corresponding RNAi plates and allowed to dry. Adult hermaphrodites were placed onto corresponding plates and allowed to lay eggs for 5 h to synchronize the F1 progeny.

### Analysis of dopaminergic neurodegeneration

*C. eleg*ans dopaminergic neurons were analyzed for degeneration as previously described^57^. Briefly, on the day of analysis, which was performed with the investigator blinded to sample identities, the six anterior dopaminergic neurons [four CEP (cephalic) and two ADE (anterior deirid)] were examined in 30 randomly selected worms, repeated three times, for a total of 90 animals. Each animal was considered normal when there was a full complement of six anterior DA neurons. However, if a worm displayed any degenerative phenotype, such as a missing dendritic process, cell body loss, or a blebbing neuronal process, it was scored as degenerating. An average of total percentage of worms with normal neurons was reported in the study. A one-way ANOVA and Tukey’s post hoc test were used for statistical analysis.

### Analysis of mitochondrial morphology in body-wall muscle cells

SJ4103 and PS6192 were utilized and scored as described previously^11, 12^. The mitochondrial matrix reporter was composed of the *myo-3* promoter ligated to a mitochondrial leader sequence fused to GFP (referred to as “mitoGFP”)^12, 58^. Adult hermaphrodites were allowed to lay eggs on corresponding treatment plates for 5 h at 20 °C and were then removed. Animals were treated with 20 × concentrations of metabolite until day of analysis, which was performed with the investigator blinded to sample identities. Mitochondrial morphology in nematodes was measured by examining a consistent region near the vulva. In all cases, between five to eight muscle cells were examined. Mitochondrial morphology was considered normal when the majority of the cells within a single worm had ordered, tubular-shaped mitochondria. Fragmented cells consisted of disorganized circular forms of mitochondria and fused mitochondria had elongated and connected formations^12^. We examined 30 worms and repeated the analysis three times, so that 90 animals were analyzed. An average percentage of worms with each mitochondrial phenotype was reported in the study. A one-way ANOVA and Tukey’s post hoc test were used for statistical analysis.

### Analysis of mitochondrial membrane potential (ΔΨ_m_)

Tetramethylrhodamine ethyl ester (TMRE; Biotium, Fremont, CA, USA) is a lipophilic cation that accumulate into the mitochondrial matrix of live nematodes based on its ΔΨ_m_^20, 21, 59^. *C. elegans* takes up TMRE from culture medium and incorporate it broadly into its tissues. Synchronized N2 (Bristol) animals were exposed to RNAi bacteria with metabolite in EtAc or EtAc alone until day 8 post-hatching. Worms were then placed on NGM plates containing 0.1μM TMRE for 18 h and washed with M9 before analysis, which was performed with the investigator blinded to sample identities. The live animals were photographed using a Nikon Eclipse E800 epifluorescence microscope at 10 × magnification. Relative uptake of the fluorescent dye was measured using MetaMorph software (Molecular Devices, Sunnyvale, CA, USA). When analyzing each animal, three 65 × 65 μm boxes were placed from most anterior portion of the intestine toward the vulva. Three independent replicates were conducted for each treatment with 30 animals per replicates for a total of 90 animals. A one-way ANOVA and Tukey’s post hoc were used for statistical analysis.

### ATP measurements

The ATP measurement assay was conducted as described previously^60^, with minor modifications. Animals were fed bacterial clones expressing EV or *eat-3* (RNAi) with metabolite in EtAc or EtAc alone from hatching until the analysis. The assay was performed with the investigator blinded to sample identities. Briefly, 100 age-synchronized young adult worms were collected in M9 buffer and washed three times. Worm pellets were treated with three freeze-thaw cycles and boiled for 15 min to releases ATP and destroy ATPase activity. Samples were then spun at 4 °C at 11000 g for 10 min. ATP contents were measured using an ATP determination kit (Molecular Probes, Eugene, OR, USA). Three independent samples were obtained for each condition and each sample was measured in duplicate using a Biotek Synergy H1 microplate reader. For normalization, protein levels from the same preparation were determined using a BCA assay (Thermo Scientific, Rockford, IL, USA).

### Quantitative Real-Time PCR

Total RNA was isolated from 100 L4 worms (N2) from each independent sample using TRI reagent (Molecular Research Center). cDNA was synthesized with iScript Reverse Transcription Supermix for qRT-PCR (Bio-Rad, Hercules, CA, USA), as previously described^11^. qRT-PCR was performed with IQ-SYBR Green Supermix (Bio-Rad) with the Bio-Rad CFX96 Real-Time System. Each reaction contained: 7.5 μl of the IQSYBR Green Supermix, 200 nM of forward and reverse primers, and 0.3 μl cDNA, to a final volume of 15 μl. Expression levels were normalized to three reference genes (*cdc-42, tba-1*, and *snb-1*) and were calculated using qBase^PLUS^ version 2.6 (Biogazelle, Gent, Belgium) for determining reference target stability. This study was performed using three technical replicates with three independent biological replicates. Each primer pair was confirmed for at least 90-110% efficiency in a standard curve on N2 cDNA. The following primers were used for the assays;

*drp-1* Forward: GAAGACGGTCAAATGGAACAC

*drp-1* Reverse: GCACGGCATCGAAGTCTGT

*fzo-1* Forward: GTGCTGCCGATAATGAACCAC

*fzo-1* Reverse: TTCCCGCTGTTCAGAACTAAC

*eat-3* Forward: CGACATCTGCTCAAACTTCGAT

*eat-3* Reverse: CCAAGACCCATTTGAATCGAAC

*ced-9* Forward: CCATCACCGAGTAGGCAG

*ced-9* Reverse: CGACCACAAATCCCTCGATA

*snb-1* Forward: CCGGATAAGACCATCTTGACG

*snb-1* Reverse: GACGACTTCATCAACCTGAGC

*tba-1* Forward: GTACACTCCACTGCTCTGCTGACAAG

*tba-1* Reverse: CTCTGTACAAGAGGCAAACAGCCATG

*cdc-42* Forward: CTGCTGGACAGGAAGATTACG

*cdc-42* Reverse: CTCGGACATTCTCGAATGAAG

### Genomic DNA isolation and mitochondrial DNA copy number determination

*C. elegans* genomic DNA was isolated as previously described^61^, with minor modification. Briefly, N2 animals at day 8 post-hatching, exposed to RNAi conditions, were washed with M9 buffer to remove bacteria. Then, 6 worms from each condition were collected in 25 μl of worm lysis buffer pre-aliquoted into PCR tubes, and frozen in liquid nitrogen immediately for 5 min. The standard thermal cycler program used in this analysis was 60 min at 65 °C, 15 min at 95 °C, and hold at 8 °C. Samples were prepared in triplicate per condition. Using the lysed worm samples, the relative copy number of mtDNA to ncDNA was measured by qRT-PCR as described previously, which is specific for *C. elegans* DNA^61^. For each sample, three biological and three technical replicates were averaged from a single real-time qPCR run. A one-way ANOVA with Tukey’s post hoc test were used for multiple comparisons.

### Statistical analysis

Statistical analysis was performed using the GraphPad Prism software (Graphpad Software, Inc., La Jolla, CA, USA) and the appropriate statistical test and post hoc tests were applied as described. Data were expressed as the mean ± S.E.M. *P *<* 0.05, **P *<* 0.01, ***P *<* 0.001 and ****P *<* 0.0001 were considered statistically significant.
